# Non-invasive sampling procedure revealing the molecular events at different abutments of bone-anchored hearing systems–A prospective clinical pilot study

**DOI:** 10.3389/fnins.2022.1058689

**Published:** 2022-11-08

**Authors:** Martin L. Johansson, Omar Omar, Margarita Trobos, Sofia Jonhede, Hanna Peters, Malou Hultcrantz, Peter Thomsen

**Affiliations:** ^1^Department of Biomaterials, Institute of Clinical Sciences, Sahlgrenska Academy, University of Gothenburg, Gothenburg, Sweden; ^2^Research and Technology, Oticon Medical AB, Askim, Sweden; ^3^Department of Biomedical Dental Sciences, College of Dentistry, Imam Abdulrahman Bin Faisal University, Dammam, Saudi Arabia; ^4^Department of Clinical Science, Intervention and Technology, Karolinska Institutet, Stockholm, Sweden

**Keywords:** abutment surface, BAHS, gene expression, bone-anchored hearing, infection, inflammation, percutaneous, Holgers score

## Abstract

**Purpose:**

To investigate the molecular activities in different compartments around the bone-anchored hearing system (BAHS) with either electropolished or machined abutments and to correlate these activities with clinical and microbiological findings.

**Materials and methods:**

Twelve patients received machined or electropolished abutments after implant installation of BAHS. Peri-abutment fluid and tissue were collected from baseline to 12 months. Gene expression of cytokines and factors related to tissue healing and inflammation, regeneration and remodelling, as well as bacterial recognition were determined using quantitative-polymerase chain reaction (qPCR). The clinical status was evaluated using the Holgers scoring system, and bacterial colonisation was investigated by culturing.

**Results:**

The gene expression of inflammatory cytokines (IL-8, IL-1β, and IL-10) and bacteria-related Toll-like receptors (2 and 4) was higher in the peri-abutment fluid than at baseline and in the peri-abutment tissue at 3 and 12 months. Conversely, the expression of genes related to tissue regeneration (Coll1a1 and FOXO1) was higher in the tissue samples than in the peri-abutment fluid at 3 and 12 months. Electropolished abutments triggered higher expression of inflammatory cytokines (IL-8 and IL-1β) (in peri-abutment fluid) and regeneration factor FOXO1 (in peri-abutment tissue) than machined abutments. Several cytokine genes in the peri-abutment fluid correlated positively with the detection of aerobes, anaerobes and *Staphylococcus* species, as well as with high Holger scores.

**Conclusion:**

This study provides unprecedented molecular information on the biological processes of BAHS. Despite being apparently healed, the peri-abutment fluid harbours prolonged inflammatory activity in conjunction with the presence of different bacterial species. An electropolished abutment surface appears to be associated with stronger proinflammatory activity than that with a machined surface. The analysis of the peri-abutment fluid deserves further verification as a non-invasive sampling and diagnostic procedure of BAHS.

## Introduction

The percutaneous bone-anchored hearing system (BAHS), first introduced 40 years ago, is now an established treatment for conductive or mixed hearing losses as well as single-sided deafness. Despite clinical success, particularly in terms of anchorage in the temporal bone by means of osseointegration, the treatment is still associated with adverse soft tissue reactions adjacent to the skin penetrating the abutment (Kiringoda and Lustig, 2013; Verheij et al., 2016; Lagerkvist et al., 2020). Hence, there is a need to understand the biological mechanisms adjacent to the skin penetrating abutment for further improvements in the treatment modality, including the system design and its maintenance (e.g., postoperative cleaning and diagnostic and treatment procedures in case of adverse reactions). The application of molecular techniques to analyse the biological events in the interface between the recipient tissues and implanted material has become a key experimental approach in different animal models. However, translating these approaches to clinical setup requires reliable, non-invasive procedures to harvest the relevant cellular compartments around, for instance, the implanted BAHS system. Such an approach may provide not only site-specific information on interfacial molecular regulation but also a tool for early diagnostics and the monitoring of adverse events at the implanted device.

Installation of a foreign material in tissue initiates a cascade of processes involving the local inflammatory response overlapped and followed by regenerative and remodelling processes (Anderson et al., 2008). These processes are mediated and orchestrated by cytokines, chemokines, matrix metalloproteinases (MMPs) and many transcription and tissue regeneration factors (Luttikhuizen et al., 2006). For example, inflammatory cells regulate the immune response and wound healing by producing pro- and anti-inflammatory cytokines such as interleukin-1beta (IL-1β), IL-8, IL-10, and tumour necrosis factor-alpha (TNF-α) (Gretzer et al., 2006; Anderson et al., 2008). Although many general mechanisms of the host response to a biomaterial have been elucidated (Klopfleisch and Jung, 2017), our understanding of the molecular interactions is still limited. Moreover, even though the molecular processes related to osseointegration have been partially revealed (Omar et al., 2011; Slotte et al., 2012), the cellular and molecular events in the soft tissue surrounding a percutaneous implant are largely not understood. A major hallmark is the permanent breach of the protective skin barrier, facilitating a continuous microbial intrusion and load to the system and the surrounding soft tissue, keeping the tissue around the abutment in a chronic state of inflammation (Holgers et al., 1995; Calon et al., 2018b). For BAHS, immune cells and microorganisms may be localised in several compartments: on the surface of the abutment, in the exudate around the abutment, and in the surrounding connective tissue (Holgers et al., 1995; Holgers and Ljungh, 1999; Grant et al., 2010; van Hoof et al., 2015).

Host responses to a biomaterial can be influenced and mediated through factors such as implant design, surface properties, anatomical location, host status, and surgical technique (Klopfleisch and Jung, 2017; Johansson, 2018). Furthermore, immune reactions may be induced by interfacial shear and strain forces between the tissue and the material (Hilborn and Bjursten, 2007). One of the key characteristics of an implant is its physicochemical and topographical nature (Rompen et al., 2006). Abutments for the BAHS are typically machined, characterised by a relatively ordered surface topography, consisting of ridges and valleys, with a surface roughness (Sa) parameter ranging between 0.5 and 2.0 μm (Albrektsson and Wennerberg, 2004). Whereas bone-anchored implants have been a major target for different surface modifications, aiming to promote bone regeneration and biomechanical fixation at the bone implant interface, less effort has been made with regard to the abutment surface interfacing with soft tissue, as in the case of skin-penetrating devices. In fact, some studies suggested that polished titanium surfaces, being smoother, are associated with lower bacterial adhesion and/or biofilm formation in contrast to rougher surfaces (Rimondini et al., 1997; Uhlmann et al., 2018). It can also be hypothesised that a smoother polished abutment surface will prevent soft tissue-to-abutment adhesion, thereby reducing the interfacial strains. However, such a comparison between machined and electropolished abutments for BAHS has been much less approached, particularly with respect to the effects on the early healing processes and bacterial accumulation after installation in human subjects.

The aims of this prospective clinical pilot study were (i) to investigate the molecular activities of different biological processes in the peri-abutment soft tissue and in the peri-abutment exudate of BAHS installed with either electropolished or machined abutments, (ii) to correlate the molecular activities of host cells with clinical and microbiological findings, and (iii) to evaluate and compare two different sampling methods (peri-abutment exudate and soft tissue).

## Materials and methods

### Study design

The study was a sponsor-initiated, single-center, prospective, controlled, pilot case series clinical investigation. The primary objective was to compare biomarkers of tissue inflammation and repair at the implant-skin interface between machined and electropolished abutments 3 months after surgery.

### Patients and implants

**FIGURE 1:**
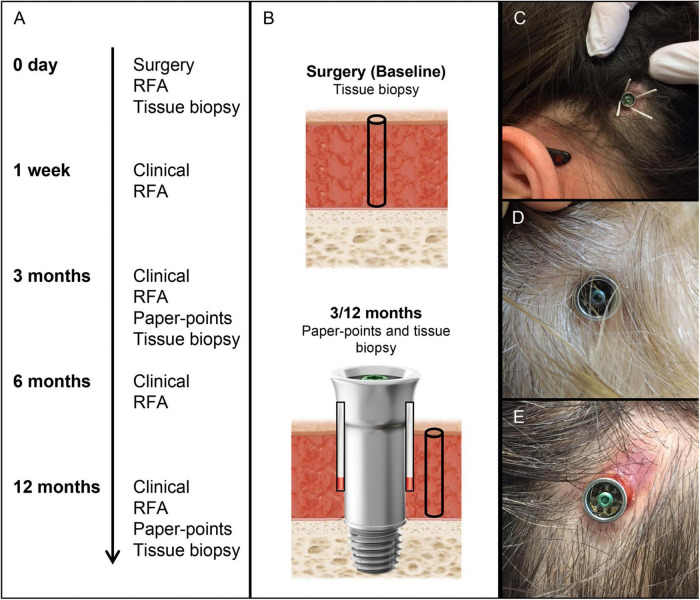
Timeline, sampling, and clinical photographs of BAHS. **(A)** Timeline and analytical procedures at each time period. **(B)** Sampling baseline tissue (biopsy for gene expression and CFU) and peri-abutment fluid (paper points for gene expression and CFU at 3 and 12 months), surrounding soft tissue (biopsy for gene expression and CFU at 3 and 12 months), and abutment (for CFU at 3 and 12 months). **(C)** Sampling of peri-abutment fluid with paper points. **(D,E)** Clinical picture of Holgers scoring 0 **(D)** and 3 **(E)** after 3 months of implantation. CFU, Colony Forming Unit; RFA, Resonance Frequency analysis.

Twelve adult patients were enrolled consecutively in the control (machined abutment, *n* = 7) and test (electropolished abutment, *n* = 5) groups. Patients were excluded if they had (i) skin thickness > 10 mm, (ii) diseases known to compromise bone quality or (iii) previous irradiation in the implant area. Surgery was performed using the Minimally Invasive Ponto Surgery (MIPS) technique (Oticon Medical, Askim Sweden) (Johansson et al., 2017; Calon et al., 2018a). The patients received the Ponto Wide implant (diameter 4.5 mm, length 4 mm), premounted with either a machined or an electropolished abutment of suitable length (diameter 5 mm, length 6, 9 or 12 mm) (Oticon Medical, Askim Sweden). Follow-up visits were performed at 1 week, 3 months and 12 months. All data were recorded in paper-based case report forms. A summary of the examinations, obtained parameters and sampling is shown in Figures 1A,B.

Both types of abutments were manufactured from commercially pure titanium grade 4, having either a machined (control) or electropolished (test) surface. Electropolishing was performed using an ElpoLux TI electrolyte (ElpoChem AG, Volketswil, Switzerland). The manufacturing, postprocessing and surface characterisation of the abutments have been previously described in detail (Trobos et al., 2018). In brief, the mean surface roughness (Sa) for the commercially available machined abutment and the electropolished abutments was 169 (25) nm and 120 nm (30) nm (*p* = 0.0007), whereas the developed interfacial area ratio (Sdr) was 82 (9.2)% and 14.8 (10.5)% (*p* < 0.0001), respectively [values presented as MEAN (SD)].

### Clinical evaluation

Baseline demographics were recorded, and at the schedule follow-up visits, clinical parameters, including the Holgers score (Holgers et al., 1988), hygiene score, pain, numbness, and implant stability, were recorded.

Baseline demographics, surgery characteristics and clinical outcomes for the two study groups have been previously reported (Trobos et al., 2018; Supplementary material). In summary, there were no significant differences in any of the clinical outcome measures (between the test and control groups after 3 and 12 months). Pooling the groups did not reveal any significant difference between 3 and 12 months in any of the clinical outcome measures (Supplementary material). In the test group, two patients had adverse skin reactions (Holgers score ≥ 2), eventually leading to removal of the abutment for one of these patients.

### Study interventions

At surgery, the skin was disinfected (ethanol, 70% w/v) prior to the administration of local anaesthesia. A skin tissue biopsy was harvested using a 1 mm biopsy punch at the predetermined retroauricular position of the abutment and preserved in RNAlater solution (Qiagen GmbH, Hilden, Germany) for baseline gene expression analysis (Figure 1). The patients were thereafter implanted with the BAHS system using the MIPS technique. At the 3- and 12-month follow-up visits, two paper points (Roeko ISO, size 45, Coltene, Langenau, Germany) were inserted in the space between the abutment and the surrounding skin (in quadrants 12 and 6 o’clock) and left for 60 s (Figure 1C). The two paper points were pooled and preserved in RNAlater solution (Qiagen GmbH) for subsequent molecular analysis. Thereafter, the skin was disinfected (ethanol, 70% w/v), and a soft tissue biopsy was collected at the border of the abutment using a 1 mm biopsy punch and preserved in RNAlater solution (Qiagen GmbH) for subsequent gene expression analysis. All samples allocated for molecular analysis were kept at 4°C until processing and analysis.

In parallel with the sampling for the molecular analysis, similar samples were harvested for microbiological evaluation at baseline (soft tissue biopsy) and after 3 and 12 months (peributment fluid, using two paper points, and soft tissue biopsy, using a tissue punch). In addition, at 3 and 12 months, the abutments were retrieved for microbial cultures. All samples allocated for microbiological analysis were preserved in ESwab™ medium (Copan Diagnostics, Murrieta, CA, USA).

### Quantitative-polymerase chain reaction

Gene expression analysis was performed on soft tissue biopsies collected at baseline as well as on cells in the peri-abutment fluid and tissue surrounding the abutment after 3 and 12 months of implantation. Total RNA was extracted from the two sample types (i.e., fluid and tissue cells). The samples were homogenised in Qiazol (Qiagen GmbH) with a steel bead (5 mm) for 5 min in a TissueLyzer (Qiagen GmbH). Chloroform was subsequently added followed by centrifugation for 15 min at 12000 g. The upper RNA phase was collected and further purified using RNeasy Micro- and Mini-kits (Qiagen GmbH) for the abutment fluid and the tissue samples, respectively. All samples were then treated with RNase-free DNase Set (Qiagen GmbH) to reduce gDNA contamination. The concentration of the purified RNA was analysed on a nanophotometer (IMPLEN GmbH, Munich, Germany), and quality control was performed using an RNA Nano chip in the Agilent 2100 Bioanalyzer (Agilent Technologies, CA, USA). The extracted RNA was normalised to 50 ng/μl and then reverse transcribed to cDNA in single 10-μl reactions with 5 μl of sample and 1 μl of TATAA RNA spike (used for the evaluation of inhibition). The reaction was performed according to the manufacturer’s instructions using a TATAA GrandScriptTM kit (TATAA Biocenter, Gothenburg, Sweden) to generate cDNA for relative quantification of mRNA. Thereafter, cDNA was diluted 10 times and stored at −20°C until further analysis.

Prior to quantitative analysis, reference gene screening was performed to determine the best stable reference gene(s). The analysis was performed in duplicate in 10 μl reactions on a qPCR cycler (CFX96, Bio-Rad Laboratories, Inc., CA, USA). The data were analysed with GenEx (MultiD Analyses AB, Gothenburg, Sweden) using the algorithms NormFinder and geNorm to select the most suitable reference genes. The screening revealed that the combination of hypoxanthine phosphoribosyltransferase 1 (HPRT) and tyrosine 3/tryptophan 5-monooxygenase activation protein zeta polypeptide (YWHAZ) provides the most stable reference for normalisation.

Primer design for the selected genes of interest was conducted in Primer Blast.^1^ The sequences for the primers are available at TATAA Biocenter. The gene panel was selected to represent important biological processes, such as inflammation: interleukin-8 (IL-8), interleukin-1beta (IL-1β), and interleukin-10 (IL-10); vascularisation: vascular endothelial growth factor (VEGF); soft tissue regeneration: collagen 1a1 (Coll1a1) and transcription factor forkhead box protein O1 (FOXO1); tissue degradation and remodelling factors: metallopeptidase 9 (MMP9) and tissue inhibitor of metalloproteinase (TIMP1); and bacterial infection-related genes: Toll-like receptors 2 and 4 (TLR2 and TLR4).

After evaluating the reference gene panel and designing and validating the panel of target genes, cDNA was subjected to quantitative polymerase chain reaction (qPCR) analysis. The analysis was performed using TATAA SYBR GrandMaster Mix (TATAA Biocenter) in 10 μL reactions on the CFX96 platform (Bio-Rad Laboratories). The thermal cycling protocol was 95°C for 30 s, followed by 40 cycles at 95°C for 5 s, 60°C for 15 s, and 72°C for 10 s. The quantities of the target genes were normalised using the mean of the reference genes HPRT and YHWAZ. The normalised quantities were calculated using the delta-Cq method and assuming 100% PCR efficiency (2^Δ^ *^Cq^*) (Pfaffl, 2001). The procedure and reporting of the qPCR gene expression analysis followed the MIQE guidelines (Bustin et al., 2009).

### Microbiological analysis

Microbiological analysis of the retrieved abutment, peri-abutment fluid (paper point) and soft tissue (biopsies) was performed within 2 days after retrieval. In brief, the total aerobic and anaerobic bacteria were determined on abutment [Colony Forming Unit (CFU)/mm^2^ abutment], paper-points (CFU/paper-points) and in soft tissue samples (CFU/biopsy). In addition, staphylococci, enterococci, *Escherichia coli*, and *Pseudomonas aeruginosa* were quantitated using selective media.

### Statistics

Statistical comparisons of gene expression were conducted between the electropolished and machined abutments for each compartment and between baseline (BL), peri-abutment tissue and peri-abutment fluid compartments for each abutment type using multivariate analysis of variance (MANOVA). Pearson correlation was conducted to evaluate associations between gene expression in the different compartments and the microbial and clinical findings. The level of significance was set at *p* < 0.05 (for the comparative analysis) and at *p* < 0.01 (for the correlation analysis). In the MANOVA, the data were adjusted with the *post-hoc* least significant difference for multiple comparisons. Statistical analysis was performed using SPSS software (Version 28.0.1, IBM Corporation, USA).

## Results

In total, 12 patients were included in the study, with the control group (*n* = 7) receiving a machined abutment and the test group (*n* = 5) receiving an electropolished abutment. During the follow-up period, two implants in the control group were lost between 3 and 12 months.

### Gene expression of inflammatory cytokines

**FIGURE 2:**
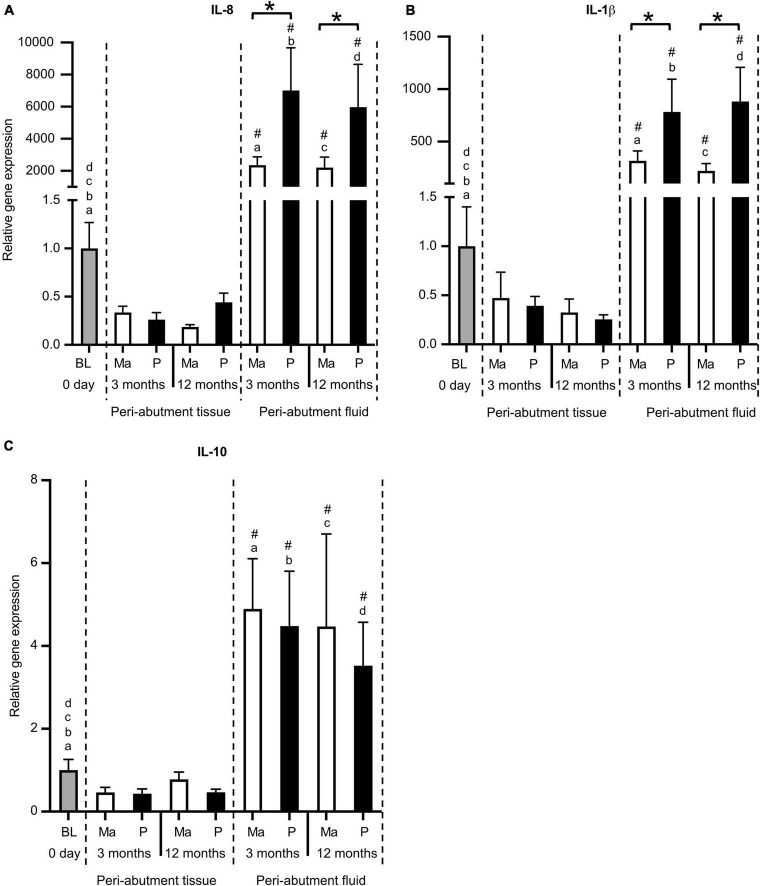
Gene expression of inflammatory cytokines. **(A)** Proinflammatory cytokine interleukin-8 (IL-8). **(B)** Proinflammatory cytokine interleukin-1beta (IL-1β). **(C)** Anti-inflammatory cytokine interleukin-10 (IL-10). The column graphs show the relative gene expression at baseline (BL) and in the peri-abutment tissue and peri-abutment fluid at machined (M) and electropolished (P) abutments after 3 and 12 months of implantation. Statistically significant differences (*p* < 0.05) between BL and either compartment (“Peri-abutment tissue” or “Peri-abutment fluid”) and between the two compartments are indicated by similar small letters and hash signs, respectively. The differences between machined and electropolished abutments for each compartment are indicated by bars and asterisks.

In general, the expression of proinflammatory cytokines (IL-8 and IL-1β) was tremendously higher in the immediate peri-abutment fluid collected by paper points than in the peri-abutment tissue or tissue collected at baseline (Figure 2). In fact, the expression of IL-8 and IL-1β was between 2000–7000-fold and 200–800-fold, respectively, higher in the peri-abutment fluid than in the peri-abutment tissue or baseline (Figures 2A,B). Similarly, the expression of the anti-inflammatory cytokine IL-10 was 4- to 5-fold higher in the peri-abutment fluid than in either of the tissue samples.

When comparing the two abutment types, 2.5–4-fold significantly higher expression of IL-8 and IL-1β was demonstrated in the peri-abutment fluid around the electropolished abutment compared with the machined abutment at 3 and 12 months (Figures 2A,B). Nonetheless, no significant differences between the two abutment types were detected in the peri-abutment tissue with respect to the inflammatory cytokines (Figures 2A,B). The anti-inflammatory cytokine (IL-10) did not show major differences between the two abutment types in either the peri-abutment fluid or the surrounding tissue (Figure 2C).

### Gene expression of Toll-like receptors

**FIGURE 3:**
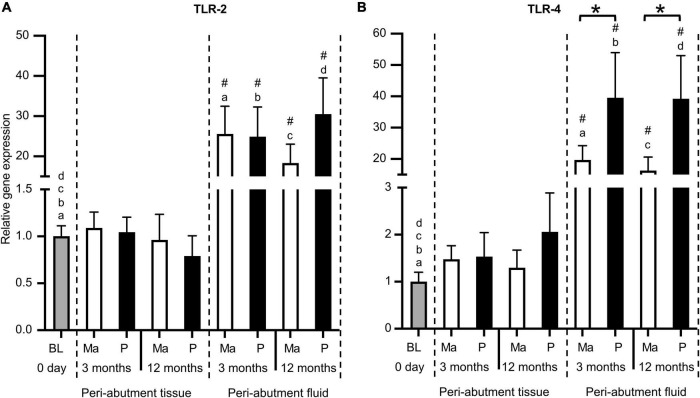
Gene expression of Toll-like receptors. **(A)** Toll-like receptor 2 (TLR2). **(B)** Toll-like receptor 4 (TLR4). The column graphs show the relative gene expression at baseline (BL) and in the peri-abutment tissue and peri-abutment fluid at machined (M) and electropolished (P) abutments after 3 and 12 months of implantation. Statistically significant differences (*p* < 0.05) between BL and either compartment (“Peri-abutment tissue” or “Peri-abutment fluid”) and between the two compartments are indicated by similar small letters and hash signs, respectively. The differences between machined and electropolished abutments for each compartment are indicated by bars and asterisks.

Similar to the inflammatory cytokines, the expression of TLR receptors 2 and 4 revealed higher abundance in the peri-abutment fluid than the constitutive tissue expression at baseline or in the surrounding peri-abutment tissue at 3 and 12 months (12–38-fold difference) (Figure 3). Comparing the two abutments, whereas no significant difference was detected for TLR-2, the expression of TLR-4 was 2-fold higher in the fluid around the electropolished abutment than that around the machined abutment at both the 3- and 12-month observation periods (Figure 3B).

### Gene expression of vascularisation and tissue regeneration

**FIGURE 4:**
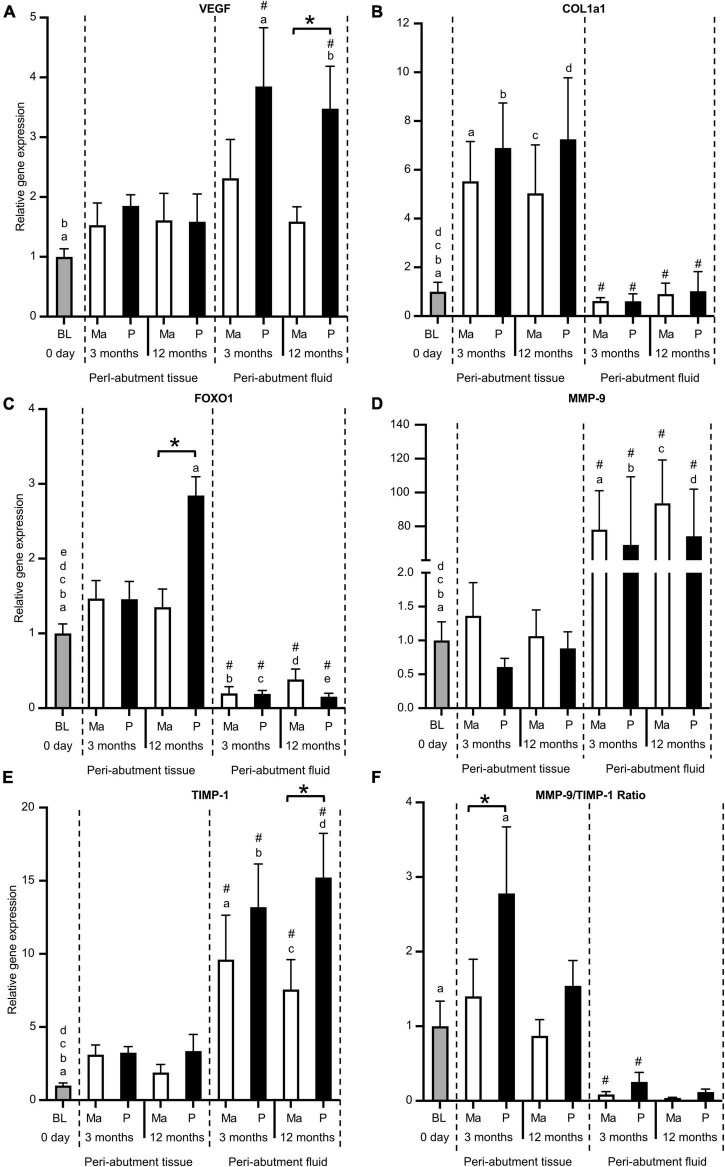
Gene expression of vascularisation, tissue regeneration, tissue degradation, and remodelling factors. **(A)** Vascularisation factor vascular endothelial growth factor (VEGF). **(B)** Extracellular matrix component collagen 1a1 (Coll1a1). **(C)** Transcription factor forkhead box protein O1 (FOXO1). **(D)** Tissue degradation enzyme matrix metallopeptidase 9 (MMP9). **(E)** Tissue inhibitor of metalloproteinase (TIMP1). **(F)** Gene expression ratio of MMP9/TIMP1. The column graphs show the relative gene expression at baseline (BL) and in the peri-abutment tissue and peri-abutment fluid at machined (M) and electropolished (P) abutments after 3 and 12 months of implantation. Statistically significant differences (*p* < 0.05) between BL and either compartment (“Peri-abutment tissue” or “Peri-abutment fluid”) and between the two compartments are indicated by similar small letters and hash signs, respectively. The differences between machined and electropolished abutments for each compartment are indicated by bars and asterisks.

For the machined abutment, VEGF expression was comparable between the peri-abutment fluid versus the surrounding tissue or baseline (Figure 4A). In contrast, polished abutment showed significantly higher VEGF expression in the peri-abutment fluid than in the surrounding tissue or at baseline (Figure 4A). Furthermore, the polished abutment demonstrated a 2-fold significantly higher VEGF expression than machined in the peri-abutment fluid. The expression of extracellular matrix component (COL1a11a1) and transcription factor FOXO1 revealed an opposite pattern to the inflammatory cytokines, where 5.5–11-fold and 8–18-fold, respectively, higher levels were detected in the peri-abutment tissue than that observed in the peri-abutment fluid (Figures 4B,C). Furthermore, whereas the expression of COL1a1 was significantly higher for all groups in peri-abutment tissue vs. baseline, the expression of FOXO1 was comparable, except for the peri-abutment tissue in the electropolished group at 12 months, which demonstrated a 2.5-fold significantly higher level than BL. The expression of FOXO1 was generally lower in the peri-abutment fluid than in BL. Comparing the two abutments, whereas no difference was found between the machined and electropolished abutments regarding the expression of Coll1a1 (Figure 4B), the expression of FOXO1 was significantly enhanced in response to the electropolished abutment vs. the machined abutment, only in the surrounding tissue after 12 months (Figure 4C).

### Gene expression of tissue degradation and remodelling factors

The expression of the tissue degradation/remodelling enzyme (MMP9) and its inhibitor (TIMP-1) was higher in the peri-abutment fluid than at baseline or in the peri-abutment tissue (Figures 4D,E). Here, the magnitude of the fold difference between the two sites was more pronounced for MMP9 than for TIMP-1. Furthermore, when comparing the MMP9/TIMP-1 expression ratio, a higher ratio was generally found in the peri-abutment tissue than in the peri-abutment fluid, but the difference was only significant at 3 months (Figure 4F). Furthermore, the MMP9/TIMP-1 ratio revealed higher values in the tissue around electropolished vs. machined abutments, and the difference was statistically significant only at 3 months.

### Microbiological evaluation

The microbiological analysis of the different compartments has previously been reported in detail (Trobos et al., 2018). In summary, anaerobic bacteria were detected in the baseline tissue, whereas anaerobes and *Staphylococcus* spp. were detected in all three compartments (soft tissue, peri-abutment fluid, and abutment) after 3 and 12 months. The peri-abutment fluid space and abutments contained significantly higher counts of anaerobes, aerobes, and staphylococci in the electropolished abutment group than in the control (machined) group. The common skin coloniser *Staphylococcus epidermidis* was identified in all patients except one (11/12), whereas the pathogen *Staphylococcus aureus* was isolated in five of the patients.

### Correlation analysis

The results from the Pearson correlation analysis of the gene expression in the cells of the retrieved samples with the microbiological and clinical findings are shown in Table 1. When correlating the baseline gene expression in the soft tissue of the recipient site with the microbial and clinical data before and/or after receiving the BAHS, the baseline expression of both proinflammatory cytokines (IL-8 and IL-1β) demonstrated a positive association with the higher CFU values of aerobes detected in baseline soft tissue samples prior to implantation (Table 1). Furthermore, the expression of both cytokines at baseline revealed a significant positive association with higher Holgers scores of inflammation and higher reported pain after 3 and 12 months of implantation. In contrast, the baseline gene expression of TLR-4 revealed significant negative correlations with the CFU values of Aerobes, Anaerobes and *Staphylococcus* spp. in the peri-abutment fluid space after 3 and 12 months of implantation (Table 1). In parallel, the baseline IL-10 expression showed a significant negative correlation with the CFU values of *Staphylococcus* spp. in the peri-abutment fluid space after 3 and 12 months (Table 1).

**TABLE 1:** Pearson correlation analysis of gene expression with microbiological and clinical findings (data pooled for the 3-month and 12-month time points).

Genes	Correlation with microbiological and clinical parameters
IL-8_Soft tissue baseline_	CFU Aerobes_Soft tissue baseline_ (*r* = 0.9; *p* < 0.0001)
	Holger_3 and 12 months_ (*r* = 0.7; *p* < 0.0001)
	Pain_3 and 12 months_ (*r* = 0.7; *p* < 0.001)
IL-1β_Soft tissue baseline_	CFU Aerobes_Soft tissue baseline_ (*r* = 0.9; *p* < 0.0001)
	Holger_3 and 12 months_ (*r* = 0.8; *p* < 0.0001)
	Pain_3 and 12 months_ (*r* = 0.6; *p* < 0.01)
TLR-4_Soft tissue baseline_	CFU Aerobes_Peri–abutment fluid 3 and 12 months_ (*r* = −0.5; *p* < 0.015)
	CFU Anaerobes_Peri–abutment fluid 3 and 12 months_ (*r* = −0.6; *p* < 0.01)
	CFU Staph_Peri–abutment fluid 3 and 12 months_ (*r* = −0.7; *p* < 0.001)
IL-10_Soft tissue baseline_	CFU Staph_Peri–abutment fluid 3 and 12 months_ (*r* = −0.6; *p* < 0.01)
IL-1β_Peri–abutment fluid 3 and 12 months_	CFU Anaerobes_Soft tissue baseline_ (*r* = 0.5; *p* < 0.01)
	CFU Aerobes_Peri–abutment fluid 3 and 12 months_ (*r* = 0.5; *p* < 0.01)
	CFU Anaerobes_Peri–abutment fluid 3 and 12 months_ (*r* = 0.5; *p* < 0.01)
	CFU Staph_Peri–abutment fluid 3 and 12 months_ (*r* = 0.5; *p* < 0.01)
	Holger_3 and 12 months_ (*r* = 0.7; *p* < 0.001)
IL-8_Peri–abutment fluid 3 and 12 months_	Holger_3 and 12 months_ (*r* = 0.5; *p* < 0.01)
Col1a1_Peri–abutment fluid 3 and 12 months_	Holger_3 and 12 months_ (*r* = 0.5; *p* < 0.01)
FOXO1_Peri–abutment fluid 3 and 12 months_	Holger_3 and 12 months_ (*r* = 0.5; *p* < 0.01)
TIMP-1_Peri–abutment fluid 3 and 12 months_	Holger_3 and 12 months_ (*r* = 0.6; *p* < 0.01)
MMP-9_Peri–abutment tissue 3 and 12 months_	Numbness_3 and 12 months_ (*r* = 0.8; *p* < 0.001)

Gene expression was analysed in cells retrieved from soft tissue samples at baseline (0 weeks) as well as the peri-abutment fluid and peri-abutment soft tissue samples at 3 and 12 months (m) after implantation of the BAHS system. The gene expression data were pooled for the 3-month and 12-month time points.

When correlating the gene expression in peri-abutment fluid after 3 and 12 months of implantation with the corresponding microbial and clinical parameters, an important finding was the significant multiple positive correlations of IL-1β expression in the peri-abutment fluid with the CFU values of aerobes, anaerobes and *Staphylococcus* spp. in peri-abutment fluid (Table 1). Furthermore, positive correlations were registered for IL-1β in the peri-abutment fluid with CFU of anaerobes in the soft tissue at baseline and with Holgers scoring after 3 and 12 months of implantation (Table 1). Similarly, the expression of IL-8, Coll1a1, FOXO1, and TIMP-1 in the peri-abutment fluid revealed a positive association with Holgers scoring of inflammation after 3 and 12 months of implantation (Table 1).

In contrast, when correlating the gene expression in the surrounding tissue with the microbial and clinical findings, only one correlation was found, showing a positive association between the gene expression of the tissue degradation enzyme MMP9 in the abutment-surrounding tissue and higher reported scores of numbness after 3 and 12 months (Table 1).

## Discussion

Analysis of the implant-tissue interface is important for understanding the cellular response to biomaterials with different surface characteristics. A prominent finding in the comparison between electropolished and machined abutments was the stronger proinflammatory activity associated with the electropolished abutments in the peri-abutment fluid compared to the abutments with machined surfaces. Interestingly, the expression of the two proinflammatory cytokines (IL-8 and IL-1β) did not vary in the peri-abutment tissue between the two abutment types, and for both abutments, it was comparable to the level detected constitutively at baseline. Taken together, these findings reflect enhanced inflammatory activity in the very close vicinity of the electropolished abutment, which does not extend distantly in the surrounding tissue. This site-specific difference also suggests that the abutment-near cells are more prone to respond to variations in the abutment surface properties and/or the microenvironment around these abutments than cells further out in the peri-implant tissue. This novel observation considering skin penetrating abutments is in line with previous observations with implant-adherent cells, in the case of bone-anchored implants (Omar et al., 2010, 2011; Sayardoust et al., 2018), as well as exudate cells, around non-skin penetrating implants in soft tissues (Suska et al., 2001, 2005). Furthermore, analogous to mucosa-penetrating oral implants, it has been shown that the cells in the peri-implant crevicular space are responsive to not only variations in the implant surface properties but also to locally/systemically altered host conditions (Sayardoust et al., 2017a,b). Considering the present finding of a substantial difference in cytokine gene expression in the peri-abutment fluid of the two abutment types, few studies have compared the inflammatory cytokine response between electropolished and machined surfaces. For instance, in the context of osseointegration and bone response, no differences were detected between electropolished and machined screw-shaped titanium implants after early (12–72 h) or late (6–28 days) periods of healing in a rat tibia model (Karazisis et al., 2017, 2021). *In vitro*, electropolished surfaces, usually included as a control group, were generally associated with less cytokine expression and/or release when compared to different scales of roughnesses produced by different titanium surface modification techniques (Refai et al., 2004; Alfarsi et al., 2014; Ariganello et al., 2018; Beheshti Maal et al., 2020). Moreover, even when using a different class of biomaterials, polytetrafluoroethylene (PTFE) membranes with increasing microporosity were found to induce higher inflammatory cytokine secretion (including IL-1β) by macrophages than smooth unstructured dense PTFE (Bota et al., 2010; Collie et al., 2011). Altogether, the available *in vivo* and *in vitro* evidence does not support the present finding that electropolished surfaces, *per se*, trigger an intense and prolonged inflammatory cytokine response, at least at the gene level. Therefore, whether such unprecedented finding is specific to the use of electropolished BAHA abutment in that recipient tissue type in humans remains to be determined.

A plausible explanation for the enhanced inflammatory activity in the peri-abutment fluid of the electropolished type is the presence of more viable anaerobes, aerobes, and staphylococci in the peri-abutment fluid of the electropolished than in the case of the machined abutment, as reported previously from this study (Trobos et al., 2018). This assumption is further supported by the concurrent upregulation of TLR-4, a member of the Toll-like receptor (TLR) family that plays fundamental roles in the innate immune response to bacteria. Although TLR-4 is not considered directly responsive to gram-positive infections, several studies have emphasised its association with the inflammatory response to different bacterial infections, including those related to gram-positive staphylococci (Stenzel et al., 2008; Chantratita et al., 2017). In fact, the correlation analysis in the present study showed significant positive associations of IL-1β, but not TLR-4, with the detection of viable anaerobes, aerobes, and staphylococci in the peri-abutment fluid, supporting the assumption of an indirect promotion of TLR-4 expression *via* other endogenous mediators (Stenzel et al., 2008; Chantratita et al., 2017), potentially triggered in association with the enhanced proinflammatory cytokine response to these bacteria.

An intriguing finding in this study was the significant upregulation of the FOXO1 gene in the peri-abutment tissue, particularly after 12 months in the electropolished type. FOXO1 is a transcription factor that plays multiple roles in regulating cell survival and death, proliferation, cell differentiation and oxidative stress responses (Vivar et al., 2016; Graves and Milovanova, 2019; Ren L. et al., 2021; Wang et al., 2022). Moreover, several studies have demonstrated its involvement in the modulation of inflammatory processes and macrophage programming (Ito et al., 2009; Yan et al., 2020; Ren J. et al., 2021). With respect to tissue healing, although some studies have linked FOXO1 to increased fibrosis under some pathological conditions (Mori et al., 2014; Chung et al., 2019), studies have emphasised its positive roles, not only as an antifibrotic factor (Xin et al., 2018; Huang et al., 2019) but also as a promoter for wound healing, *via* coordination with TGF-β signalling, enhancement of keratinocyte proliferation and wound epithelization and remodelling processes *via* the regulation of MMP/TIMP activities (Ponugoti et al., 2013; Hameedaldeen et al., 2014; Zhang et al., 2017; Miao et al., 2019; Rao et al., 2021). Nonetheless, although the present study does not provide histological evidence of a similar or dissimilar pattern of tissue healing and regeneration between the two abutment types, the clinical evaluation did not reflect macroscopically superior healing at the electropolished abutment being associated with higher expression of FOXO1. Although speculative, being rather lately upregulated (after 12 months) at the electropolished surface, a potential regulatory role for FOXO1 on the inflammatory cytokine response, observed at this time point, cannot be excluded. Support for this assumption comes from an *in vivo* study revealing that FOXO1 plays a decisive role in promoting proinflammatory (M1) macrophage polarisation and upholding a potent adaptive immune response against *S. aureus* infection in a rat liver model (Wang et al., 2016). To this point, despite the evident molecular and microbiological differences between the electropolished and machined abutments, particularly in the peri-abutment fluid compartment, the macroscopic clinical picture did not differ between the BAHAs with either abutment type. Yet, positive associations were demonstrated for the expression of the potent cytokine IL-1β with the detection of anaerobes, aerobes, and staphylococci in the peri-abutment fluid and for the expression of all analysed cytokines (IL-1b and IL-8) and soft tissue regenerative genes (COL1a1 and FOXO1) with the clinical scoring (Holgers) results. Taken together, these findings suggest an ongoing, subclinically mounted inflammatory response in the narrow space between the electropolished abutment and the recipient tissue, which is modified by the accumulation of one or more types of bacterial species, including staphylococci in that compartment. Being largely limited to peri-abutment fluid and not extended to the surrounding tissue and not manifested clinically, it is plausible to speculate that this mounted inflammatory response succeeded in circumventing the progress of bacterial intrusion into a deeper infection. Indeed, further studies are required to test this assumption, including more insight into the role of FOXO1 in inflammatory and regenerative processes at the tissue interface with skin-penetrating devices.

### Associations between clinical and cellular response

A limited number of studies evaluating the biological processes of clinically used BAHSs have been reported (Grant et al., 2010; Calon et al., 2018b). Grant and coworkers used periostrips to collect, quantify and analyse the peri-abutment fluid around BAHS from 10 patients without inflammation and 10 with inflammation (Holgers ≥ 2) (Grant et al., 2010). The volume of the exudate was associated with the Holgers score, with higher volumes of exudate collected from cases with adverse soft tissue reactions. The inflammatory biomarkers Il-1β, Il-6, Il-8, and TNF-α and tissue metabolism biomarkers (TIMPs and MMP9) were detected in all patients irrespective of the Holgers score, with a significantly higher abundance in patients with inflammation than in those without inflammation. Furthermore, the expression of Il-1β, Il-6, TIMP2, and MMP9 was significantly correlated with inflammation, as determined by the Holgers score. In agreement with Grant, the present study found that adverse soft tissue reactions, manifested clinically by observed increased Holgers and pain scores, were associated with increased expression of the proinflammatory cytokines Il-8 and Il-1β in the peri-abutment fluid. In contrast to the present study, where new patients were implanted using a minimally invasive tissue preservation technique, the study by Grant evaluated established BAHS patients (with unknown follow-up time), and although not reported, the implants were likely installed using a tissue reduction technique. In this study, we determined the relative gene expression of the biomarkers within the cells. The influence of these observed differences in gene expression on the actual cellular response is not known.

In a clinical study of patients with BAHS using tissue preservation techniques, small soft tissue biopsies were harvested at baseline, 3 months and at the incidence of inflammation (defined as a Holgers score of ≥ 2) and subjected to qPCR analysis to determine the mRNA expression of various cytokines and growth factors (Calon et al., 2018b). In support of our findings, upregulation of the inflammatory cytokines Il-1β and Il-8 at 3 months compared with baseline coupled was observed, with an ongoing remodelling process manifested by upregulation of MMP-9 and TIMP1. Moreover, in agreement with our observations, Il-1β was associated with inflammation.

Despite the difference in microbiota and tissue, there are similarities between BAHS and the dental implant with its peri-abutment sulcus (Ghassib et al., 2019). The association between periodontitis and elevated levels of Il-1β in gingival crevicular fluid has been reported (Jaedicke et al., 2016). The higher expression of Il-1β over time (3–12 months) could be associated with the increased colonisation of aerobic bacteria from baseline to 3–12 months (Trobos et al., 2018). Hence, Il-1β may be an important biomarker in relation to both adverse soft tissue reactions and pain associated with BAHS and could potentially serve as a biomarker that might help to discriminate peri-implant health from disease. Importantly, the present study identified a correlation between the expression of proinflammatory markers in the soft tissue at baseline and subsequent adverse soft tissue reactions. Analysing the peri-abutment exudate could therefore be suggested as a non-invasive diagnostic tool to identify patients prone to adverse reactions and monitor the state of peri-abutment tissue and may be helpful in the development of host-targeted preventive and therapeutic strategies.

### Bacteriological outcome

The bacterial communities on the human scalp and scalp hair vary between individuals and are distinguishable from those of other human skin microbiomes (Watanabe et al., 2020). It has been found that distinct commensal microbiota induce IL-1β after intestinal injury, which is controlled *via* the NLRP3 inflammasome (Seo et al., 2015). In this study, the expression of the proinflammatory cytokine IL-1β at 3 and 12 months positively correlated with the presence of anaerobic bacteria in tissue at baseline. Moreover, the expression of the anti-inflammatory cytokine IL-10 in tissue at baseline correlated negatively with the subsequent presence of *Staphylococcus* spp. in peri-abutment fluid at 3 and 12 months.

Due to the prophylactic disinfection of skin prior to surgery, only one patient was colonised with very low amounts of aerobic bacteria at baseline (Trobos et al., 2018). The presence of these aerobes correlated with increased expression of IL-8 and IL-1β at baseline. A thorough skin disinfection of the patient before surgery will decrease or completely eradicate aerobes and therefore could potentially decrease the risk of proinflammatory cytokine secretion. Furthermore, at 3 and 12 months, IL-1β expression correlated with the presence of all types of detected bacteria in the peri-abutment fluid. Microbial presence may have been a main stimulus that elicited the sustained expression of this pro-inflammatory cytokine. IL-1β protects against infection by rapid recruitment of neutrophils, induction of fever, cytokines and chemokines, and adaptive immunity (Sahoo et al., 2011). Compared to other proinflammatory cytokines, IL-1β has one of the highest potential to cause host tissue damage, and various cellular mechanisms exist to control its activity.

Particularly, after 3 and 12 months of implantation, the peri-abutment fluid contained approximately 10^3^ CFU and included mainly *S. epidermidis* and *S. aureus* species (Trobos et al., 2018). It has been demonstrated that phagocytosis of the peptidoglycan from the cell wall of *S. aureus* can stimulate secretion of IL-1β. Bacteria obtain access to the deeper layers of the patient’s skin due to the percutaneous post (Broekhuizen et al., 2010). Since a breach of skin barrier cannot be avoided with percutaneous BAHS, these results stress the need for good hygiene methods and patient compliance, as well as novel antimicrobial surface approaches.

Limitations exist in this study. Being a pilot study performed in a single clinical centre and built on a relatively small number of samples, the results and conclusions require confirmation in a future, large multicentre study. Due to the relatively small sampling volumes, the current lack of data on cell phenotypes and protein expression will require larger sampling volumes. Furthermore, a global genome analysis would be an additional analytical strategy. A major strength of this study is that it was conducted prospectively with predefined exclusion criteria and using unified surgical and sampling procedures at predetermined time points. Moreover, the correlational analyses of the molecular, microbiological, and clinical outcomes compensated for the small sample size, indicating a translational opportunity to use the combinatorial, non-invasive sampling approach for monitoring the performance of BAHS.

## Conclusion

Within the limited number of patients, the sampling and subsequent analyses of the peri-abutment fluid and tissue compartments provided detailed information on the biological processes around BAHS in humans. Although the BAHS were macroscopically normally healed and functionally active, the peri-abutment fluid space of BAHS harbours prolonged inflammatory activity in conjunction with the presence of different bacterial species. Abutments with electropolished surfaces appear to be associated with stronger pro-inflammatory activity, both in the peri-abutment fluid and in the surrounding tissue, compared to abutments with machined surfaces. The sampling and molecular and microbial analyses of the peri-abutment fluid deserve further verification as a novel non-invasive diagnostic and monitoring procedure of the biological status around BAHS.

## Data availability statement

All data generated or analyzed during this study are included in this manuscript. Raw data files are available from the corresponding author upon reasonable request.

## Ethics statement

This study was approved by the Regional Ethical Review Board of Stockholm, Sweden (2014/1566-452 31/2) and was performed in accordance with the Declaration of Helsinki (Washington, 2002) and ISO 14155:2011 (Clinical Trial Registration: www.ClinicalTrials.gov, identifier: NCT02304692). Informed consent was obtained from all individual participants included in the study.

## Author contributions

MJ, SJ, HP, and MH designed and executed the clinical data collection. MH performed the surgeries and sampling procedures. SJ and MJ analysed the clinical data. MT collected and analysed the microbial culture data. MJ, OO, MT, and PT analysed the gene expression data and correlations and wrote the manuscript with input from SJ, HP, and MH. All listed authors made a significant contribution to the conception and design of the study and reviewed and approved the final manuscript.

## Footnotes

1
http://www.ncbi.nlm.nih.gov/tools/primer-blast
