# Supplementary material for: Non-invasive sampling procedure revealing the molecular events at different abutments of bone-anchored hearing systems–A prospective clinical pilot study
Source: Front Neurosci. 2022 Nov 8;16:1058689. doi: 10.3389/fnins.2022.1058689 (PMC9683095; doi:10.3389/fnins.2022.1058689)
Supplement: Supplementary file 1 [file Data_Sheet_1.PDF]

Supplementary Material 1

Baseline demographics, surgery characteristics and clinical outcome. *p*-values calculated with Fisher's exact test. Hygiene is defined as amount of debris around the abutment. ISQ; Implant stability quotient measured using Resonance Frequency Analysis (RFA).

Baseline demographics		Machined (n=7)	Electropolished (n=5)	
**Age** (years); n (SD)		64.4 (25.1)	46.2 (24.1)	
**Gender**; n (%)	Male	4 (57.1)	2 (40.0)	
	Female	2 (28.6)	3 (60.0)	
**Type of hearing loss**; n (%)				
	Acquired conductive/mixed hearing loss	3 (42.9)	3 (60.0)	
	Single sided deafness	2 (28.6)	1 (20.0)	
	Congenital conductive hearing loss	2 (28.6)	1 (20.0)	
**Smoking**; n (%)	No smoking	7 (100.0)	5 (100.0)	
	Smoking	0 (0.0)	0 (0.0)	
**Body Mass Index**; n (SD)		27.4 (6.2)	29.1 (11.2)	
**Surgery characteristics**		**Machined (n=7)**	**Electropolished (n=5)**	
**Skin thickness**; millimetres (SD)		6.4 (1.9)	6.7 (2.6)	
**Abutment length**; n (SD)	6	1 (14.3)	0 (0.0)	
	9	5 (71.4)	3 (60.0)	
	12	1 (14.3)	2 (40.0)	
**Clinical outcomes per group**		**Machined**	**Electropolished**	***p***
**Maximum Holgers scoreper patient across all visits 0-12 months**; n (%)	Grade 0	5 (71.4)	2 (40.0)	
	Grade 1	2 (28.6)	1 (20.0)	
	Grade 2	0 (0.0)	1 (20.0)	
	Grade 3	0 (0.0)	1 (20.0)	
	Grade 4	0 (0.0)	0 (0.0)	0.470
**Holgers score across all patients and all visits, 0-12 months**; n (%)	Grade 0	21(91.3)	13 (68.4)	
	Grade 1	2 (8.7)	3 (15.8)	
	Grade 2	0 (0.0)	1 (5.3)	
	Grade 3	0 (0.0)	2 (10.5)	
	Grade 4	0 (0.0)	0 (0.0)	0.168
**ISQ Low at baseline**; ISQ (SD)		49.6 (10.9)	51.2 (3.3)	0.81
**ISQ Low increase at 12 months**; ISQ (SD)		8.4 (5.7)	2.3 (11.5)	0.54

Clinical outcomes pooled		3 months	12 months	*p*
**Holgers score**; n (%)	Grade 0	9 (75.0)	7 (77.8)	
	Grade 1	2 (16.7)	1 (11.1)	
	Grade 2	0 (0.0)	1 (11.1)	
	Grade 3	1 (8.3)	0 (0.0)	
	Grade 4	0 (0.0)	0 (0.0)	0.869
**Hygiene**; n (%)	None	6 (50.0)	5 (55.6)	
	Minimal	4 (33.3)	3 (33.3)	
	Moderate	2 (16.7)	1 (11.1)	
	Abundant	0 (0.0)	0 (0.0)	1.00
**Pain**; n (%)	None (VAS = 0)	7 (58.3)	9 (100.0)	
	Limited (>0 VAS ≤3)	4 (33.3)	0 (0.0)	
	Moderate (>3 VAS ≤7)	1 (8.3)	0 (0.0)	
	Extensive (>7 VAS ≤ 10)	0 (0.0)	0 (0.0)	0.060
